# Rheology-Guided Material Extrusion of Highly Filled Sm_2_Fe_17_N_3_ and SrFe_12_O_19_ Magnetic Filaments Using a Polyolefin-Based Binder

**DOI:** 10.3390/ma19163444

**Published:** 2026-08-14

**Authors:** Muhammad Shahid Arshad, Mahrukh Sadaf, Mohor Mihelčič, Spomenka Kobe, Boris Saje, Bor Arah, Fabian Burkhardt, Ema Žagar, Rožle Repič, Lidija Slemenik Perše, Kristina Žužek

**Affiliations:** 1 Department for Nanostructured Materials, K7, Jožef Stefan Institute, Jamova Cesta 39, 1000 Ljubljana, Slovenia; spomenka.kobe@ijs.si (S.K.); boris.saje@kolektor.com (B.S.); tina.zuzek@ijs.si (K.Ž.); 2 Laboratory of Experimental Mechanics, Faculty for Mechanical Engineering, University of Ljubljana, Aškerčeva Ulica 6, 1000 Ljubljana, Slovenialidija.slemenik.perse@fs.uni-lj.si (L.S.P.); 3 Kolektor Mobility, Vojkova ulica 10, 5280 Idrija, Slovenia; 4 Center for Electron Microscopy and Microanalysis, Jožef Stefan Institute, Jamova Cesta 39, 1000 Ljubljana, Slovenia; 5 National Institute of Chemistry, KI, Hajdrihova 19, 1000 Ljubljana, Slovenia; 6 Slovenian National Building and Civil Engineering Institute, ZAG, Dimičeva ulica 12, 1000 Ljubljana, Slovenia

**Keywords:** material extrusion, magnetic filament, bonded magnets, rheology, polyolefin binder, SmFeN, SrFe_12_O_19_, additive manufacturing, highly filled feedstock

## Abstract

Highly filled magnetic filaments require binder systems that balance powder loading, melt flow, filament strength, and strand stability during material extrusion (MEX). Here, a polyolefin-based binder composed of a thermoplastic elastomer and grafted polyolefin (TPE + gPO) was used to prepare Sm_2_Fe_17_N_3_- and SrFe_12_O_19_-based magnetic filaments. The feedstocks were compounded, extruded into 2.85 mm filaments using a single-screw extrusion line with automatic spooling, and printed by MEX into ring-shaped specimens. Thermogravimetric analysis confirmed high powder contents, with residual masses of approximately 89 wt.% for Sm_2_Fe_17_N_3_ and 85 wt.% for SrFe_12_O_19_. The filaments showed good dimensional control, with a diameter of 2.85 ± 0.07 mm and ovality ≤ 0.017 mm. Small-amplitude oscillatory rheology at 200–240 °C showed pronounced shear-thinning behavior and an elastic-dominant response (G′ > G″) over 0.01–100 Hz, supporting flow through the nozzle and shape retention after deposition. Mechanical testing confirmed sufficient handling performance, with a maximum flexural stress of 36.1 ± 0.1 MPa for SrFe_12_O_19_ and a flexural modulus up to 5.5 ± 0.2 GPa for Sm_2_Fe_17_N_3_. The results demonstrate that the TPE + gPO binder provides a suitable processing window for highly filled magnetic filaments for MEX-printed bonded magnets.

## 1. Introduction

Polymer-bonded permanent magnets produced by material extrusion (MEX), also known as fused filament fabrication (FFF), offer design freedom, rapid prototyping, and low tooling costs for applications such as motors, sensors, and customized magnetic assemblies [1,2,3,4,5,6]. Several additive manufacturing (AM) routes have been investigated for bonded magnets, including binder jetting [7], stereolithography [8], selective laser sintering [9], direct-write inks [10], and MEX [3]. These techniques differ in achievable solids loading, dimensional accuracy, surface quality, density, and processing throughput [1,2,5,11]. Among them, MEX is particularly attractive because it uses relatively simple equipment and can process highly filled thermoplastic feedstocks into near-net-shape bonded magnets. However, successful MEX printing of magnetic composites requires a careful balance between high powder loading, filament flexibility, melt flow, and strand stability after deposition.

Polymer-bonded magnets are composite materials in which magnetic powders are dispersed in a polymer matrix. Common binder materials include polyamides (PAs), polypropylene (PP), polyphenylene sulfide (PPS), thermoplastic polyurethane (TPU), low-density polyethylene (LDPE), and high-density polyethylene (HDPE) [1,2,3,4,11,12]. In highly filled MEX feedstocks, the magnetic powder provides the functional magnetic response, whereas the polymer binder controls processability, filament handling, and interlayer bonding during printing [13]. Increasing the filler content is desirable for improving the magnetic powder fraction, but it also increases viscosity and can make filaments brittle or difficult to feed through the printer. Therefore, the binder must provide sufficient flexibility for spooling and feeding, while maintaining adequate stiffness to avoid buckling during printing [14]. Elastomers and polyolefin-based components are often used to improve flexibility and flow behavior in highly filled feedstocks [15,16].

The rheological behavior of highly filled magnetic filaments is critical for material extrusion processing [17]. During MEX, the feedstock must flow through the nozzle under high shear, but the deposited strand must retain its shape after extrusion. This requires a suitable viscosity profile and viscoelastic response within the printing temperature range. The melt behavior is influenced by powder loading, particle size and morphology, binder composition, powder–binder interactions, temperature, and shear rate. In highly filled systems, particle agglomeration, insufficient binder wetting, or void formation can also affect melt flow, mechanical strength, and the quality of printed parts. For this reason, rheological characterization should be considered together with morphological and mechanical analysis when developing printable magnetic filaments.

In this study, highly filled magnetic filaments based on samarium iron nitride (Sm_2_Fe_17_N_3_) and strontium ferrite (SrFe_12_O_19_) powders were prepared using a laboratory-developed polyolefin-based binder formulation composed of a thermoplastic elastomer and grafted polyolefin (TPE + gPO). These two powders were selected to evaluate the processing behavior of compositionally different magnetic fillers within the same binder system. The feedstocks were compounded, extruded into filaments, and printed into ring-shaped specimens using MEX. The powder content was determined by thermogravimetric analysis (TGA), while scanning electron microscopy (SEM) was used to assess powder dispersion, binder coverage, and void formation. Tensile and three-point bending tests were performed to evaluate filament handling and feeding performance, and oscillatory rheology was used to determine the melt behavior over the relevant processing temperature range. The aim of this work is to establish a rheology-guided processing window for highly filled Sm_2_Fe_17_N_3_- and SrFe_12_O_19_-based magnetic filaments and to assess their suitability for MEX-printed bonded magnets.

## 2. Materials and Methods

### 2.1. Materials and Preliminary Characterizations

**Figure 1:**
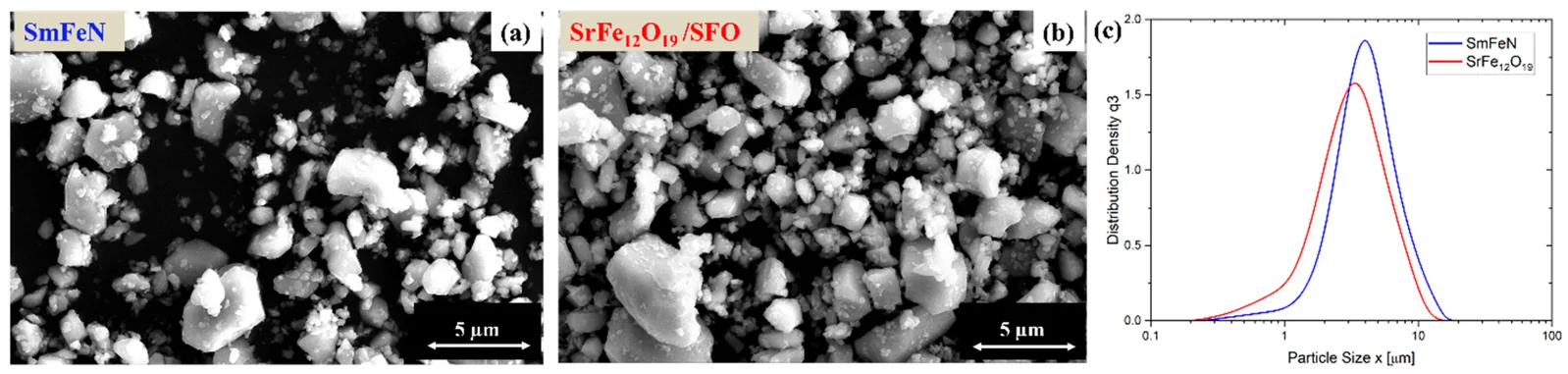
Starting magnetic powders: SEM images of (**a**) SmFeN and (**b**) SrFe_12_O_19_/SFO; (**c**) particle size distributions of SmFeN and SrFe_12_O_19_ measured by laser scattering.

The magnetic powders used in this study were samarium iron nitride powder; Sm_2_Fe_17_N_3_, hereafter denoted as SmFeN (Nichia Corporation, Tokushima, Japan); and strontium ferrite powder, SrFe_12_O_19_, hereafter denoted as SFO (OP71, DOWA Electronics Materials Co., Tokyo, Japan). According to the supplier data and particle size distribution (PSD) analysis, the SmFeN powder had a median particle size D50 of approximately 4.0 µm and a density of 7.2 g/cm^3^, while the SFO powder had a D50 of approximately 3.1 µm and a density of 5.1 g/cm^3^. The PSD were measured using laser diffraction (HELOS/BR, Sympatec GmbH, Clausthal-Zellerfeld, Germany) with R1 (0.18–35 μm) and R3 (0.9–175 μm) lenses. The results were analysed using the FREE method. Powder morphology was examined by scanning electron microscopy (SEM; Apreo 2S, Thermo Fisher Scientific, Waltham, MA, USA). Representative SEM images and PSD curves are shown in Figure 1a–c.

Both powders consisted mainly of irregular, angular particles with fine particles and larger micrometer-scale fragments. The PSD curves showed particle sizes in the few-micrometer range. SmFeN exhibited a slightly larger D50 and a narrower main distribution, whereas SFO showed a finer overall particle size and a broader distribution with a more pronounced fine-particle fraction. The measured particle size parameters are summarized in Table 1.

**Figure 2:**
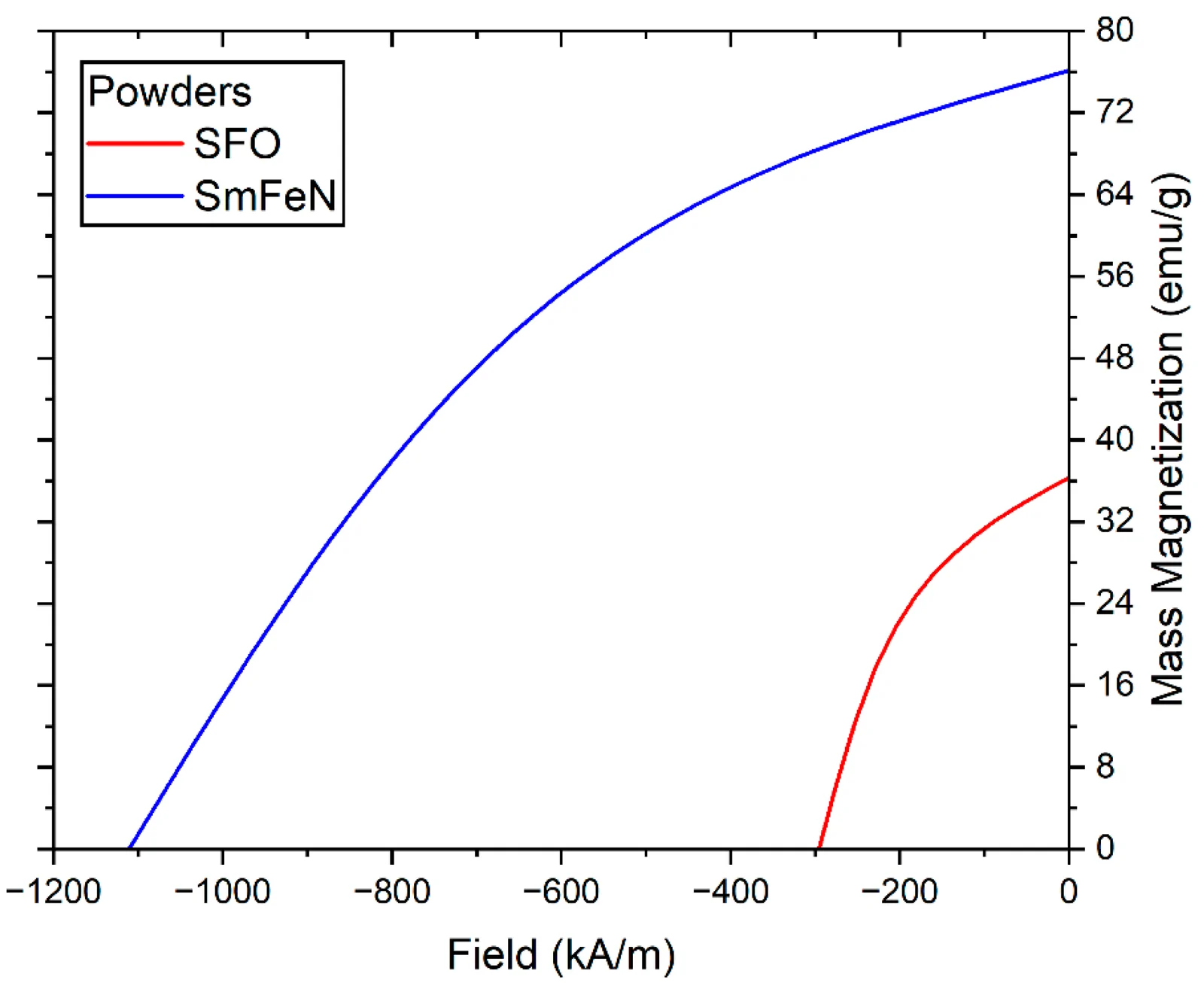
Second-quadrant VSM demagnetization curves of the starting SmFeN and SFO powders. Magnetization is reported as mass magnetization normalized by sample mass.

Magnetic characterization of powders, filaments, and selected printed specimens was performed using vibrating-sample magnetometry (VSM, Lakeshore 7304, Westerville, OH, USA) to track the evolution of mass-normalized magnetic properties during processing. The magnetization signal was normalized by the measured sample mass and reported as mass magnetization in emu/g. From the second quadrant of the VSM demagnetization curves, the coercivity, H_c_, and remanent magnetization, M_r_, were determined. The magnetic properties of the starting powders are shown in Figure 2 and summarized in Table 1.

The VSM curves show clear differences between the two powders. SmFeN exhibited a higher remanent magnetization, M_r_, of approximately 78 emu/g and a coercivity, H_c_, of approximately 1110 kA/m. SFO showed a lower M_r_ of approximately 36 emu/g and an H_c_ of approximately 296 kA/m. These values confirm the hard-magnetic character of both powders and provide the reference state for evaluating changes after compounding, filament extrusion, and MEX printing.

### 2.2. Compounding of Feedstock

A flexible commercial thermoplastic elastomer (TPE; Kraiburg TPE GmbH & Co. KG, Waldkraiburg, Germany) was used as the main elastomeric binder component, while a grafted polyolefin compatibilizer/backbone polymer (gPO; BYK Chemie GmbH, Wesel, Germany) was used as the polyolefin component. Two highly filled magnetic feedstocks were prepared using the same binder system and different magnetic powders, namely SmFeN and SFO. The feedstocks were designed to contain approximately 50 vol.% magnetic powder, corresponding to high powder contents in the range of approximately 85–90 wt.%, depending on the powder density.

The SmFeN-based feedstock, denoted as F1_Sm, contained 89.28 wt.% SmFeN powder, 7.13 wt.% TPE, and 3.59 wt.% gPO. The SFO-based feedstock, denoted as F2_Sr, contained 84.89 wt.% SFO powder, 10.06 wt.% TPE, and 5.05 wt.% gPO. The higher powder weight fraction in the SmFeN feedstock results from the higher density of SmFeN compared with SFO.

Compounding was carried out using an internal mixer equipped with counter-rotating roller rotors (HAAKE Rheomixer R3000p, Thermo Fisher Scientific Inc., Waltham, MA, USA). The mixing chamber had a volume of 310 cm^3^, and approximately 80% of the chamber volume was filled to promote efficient mixing. The binder components were added during the first minute of mixing, followed by powder addition after 3 min. The powder was added in four portions at 5 min intervals to allow torque stabilization and improve powder dispersion in the binder matrix. The compounding temperature was 200 °C, and the total kneading time was 45 min. The rotor speed was initially set to 30 rpm and was increased to 60 rpm during the final 20 min of compounding. The composition and compounding parameters of the feedstocks are summarized in Table 2. Because prolonged residence at elevated temperature can promote polymer aging or degradation, the selected kneading conditions were evaluated together with the thermal results; the processing temperature remained below the onset of the main mass-loss region observed for the binder-containing feedstocks.

After compounding, the feedstock was removed from the mixing chamber and cooled to room temperature. The solidified material was pelletized using a cutting mill (Retsch SM200, Retsch GmbH, Haan, Germany) equipped with a 4 mm × 4 mm square-opening sieve. The resulting pellets were approximately 2–3 mm in length and were dried at 50 °C for 24 h before filament extrusion.

### 2.3. Feedstock Filament Extrusion

**Figure 3:**
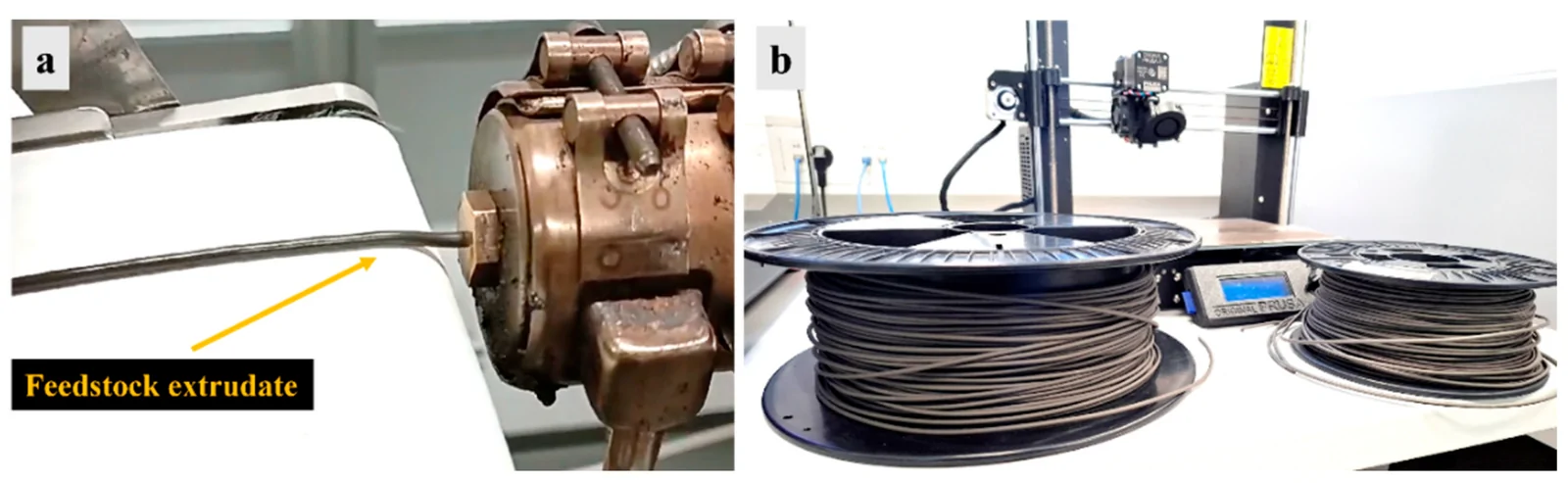
Filament extrusion and collection: (**a**) extruded F1_Sm feedstock filament transported on the conveyor belt after exiting the die and (**b**) automatically spooled feedstock filaments.

Feedstock filaments were produced using a single-screw extruder (FT-E20T-MP-IS, Dr. Collin GmbH, Maitenbeth, Germany) equipped with three heating zones. A die with a diameter of 2.85 mm was used to obtain filaments suitable for MEX printing. The extruded filament was transported on a conveyor belt, passed through a haul-off unit, and monitored continuously using a laser diameter-measuring system (Diagnostic Laser 2000, Sikora AG, Bremen, Germany). The laser system was used to record filament diameter and ovality during extrusion. An automatic spooling unit was used to collect the filaments continuously, as shown in Figure 3.

The extrusion temperature and haul-off conditions were adjusted for each feedstock to obtain a stable filament diameter. The extrusion parameters and measured filament properties are summarized in Table 3. Both feedstocks were successfully extruded and automatically spooled without filament breakage, indicating sufficient filament strength for handling after extrusion. The measured filament diameters were close to the target value of 2.85 mm, with average diameters of 2.85 ± 0.07 mm for F1_Sm and 2.85 ± 0.05 mm for F2_Sr. The corresponding ovality values were 0.017 ± 0.010 and 0.016 ± 0.009, respectively.

### 2.4. Thermal Characterization

Thermogravimetric analysis (TGA) was performed on the extruded feedstock filaments to determine the magnetic powder content and evaluate the thermal stability of the binder system. Measurements were carried out using a Mettler Toledo TGA-MS instrument (Mettler Toledo, Greifensee, Switzerland). Samples with masses between 3 and 7 mg were heated from 25 to 600 °C at a heating rate of 10 K min^−1^ under a nitrogen atmosphere with a gas flow rate of 50 mL min^−1^. The residual mass after thermal decomposition of the polymer binder was used to estimate the magnetic powder content of each filament.

Differential scanning calorimetry (DSC) was used to investigate the thermal transitions of the composite filament samples using a Mettler Toledo DSC 1 instrument (Mettler Toledo, Greifensee, Switzerland). The samples were heated from −80 to 300 °C at a heating rate of 10 K min^−1^, held briefly at 300 °C, and then cooled to −80 °C at a cooling rate of 200 K min^−1^. After an isothermal hold at −80 °C for 15 min, a second heating scan to 300 °C was performed at 10 K min^−1^. Transition temperatures and peak integrals were determined using STARe software Eval V16.40.

### 2.5. Rheological Analysis of Feedstocks

Rheological characterization of the feedstock pellets was performed using an MCR 302 rotational rheometer (Anton Paar, Graz, Austria) equipped with a parallel-plate geometry. Measurements were carried out at 200, 220, and 240 °C under an inert nitrogen atmosphere. A 25 mm diameter plate and a 1 mm measurement gap were used. The feedstock pellets were placed directly on the lower plate and allowed to melt before testing.

Amplitude sweep tests were first performed at a constant frequency of 1 Hz by applying shear stress from 10 Pa to 30 kPa to determine the linear viscoelastic region. Frequency sweep tests were then conducted within the linear viscoelastic region over a frequency range of 100 to 0.01 Hz. Depending on feedstock composition and temperature, the applied shear stress for the frequency sweep was selected between 100 and 1000 Pa. In addition, rotational flow tests were performed using a logarithmic shear-stress ramp from 100 Pa to 10 or 30 kPa. Data points were recorded every 2 s during the flow measurements.

### 2.6. Morphology and Microstructure Characterization

The morphology and microstructure of the extruded filaments and MEX-printed specimens were examined using scanning electron microscopy (SEM; Apreo 2S, Thermo Fisher Scientific, Waltham, MA, USA). The SEM was equipped with an Oxford Instruments AZtecLive Ultim Max 100 mm^2^ silicon drift detector (High Wycombe, UK) for energy-dispersive X-ray spectroscopy (EDS). Prior to analysis, the samples were embedded in resin, polished to expose the cross-sections, and carbon-coated to reduce charging during imaging and EDS analysis.

Fracture surfaces after tensile testing were examined by SEM using an ETD detector (SEM; Apreo 2S, Thermo Fisher Scientific, Waltham, MA, USA) at 20 kV. Images were collected at low and high magnifications to evaluate the macroscopic fracture surface and local filler–matrix failure features.

Secondary electron (SE) and backscattered electron (BSE) images were acquired at different magnifications using an accelerating voltage of 20 kV. Imaging was performed at multiple positions across the filaments and printed specimens, including the center and outer regions, to assess particle dispersion, binder distribution, voids, and interfacial quality. Because of the magnetic nature of the samples, additional stigmation correction was required during imaging.

### 2.7. Mechanical and Physical Characterization of Filaments

The mechanical behavior of the feedstock filaments was evaluated by three-point bending and tensile testing under standard laboratory conditions (23 °C and 50% relative humidity). Three specimens were tested for each feedstock.

**Figure 4:**
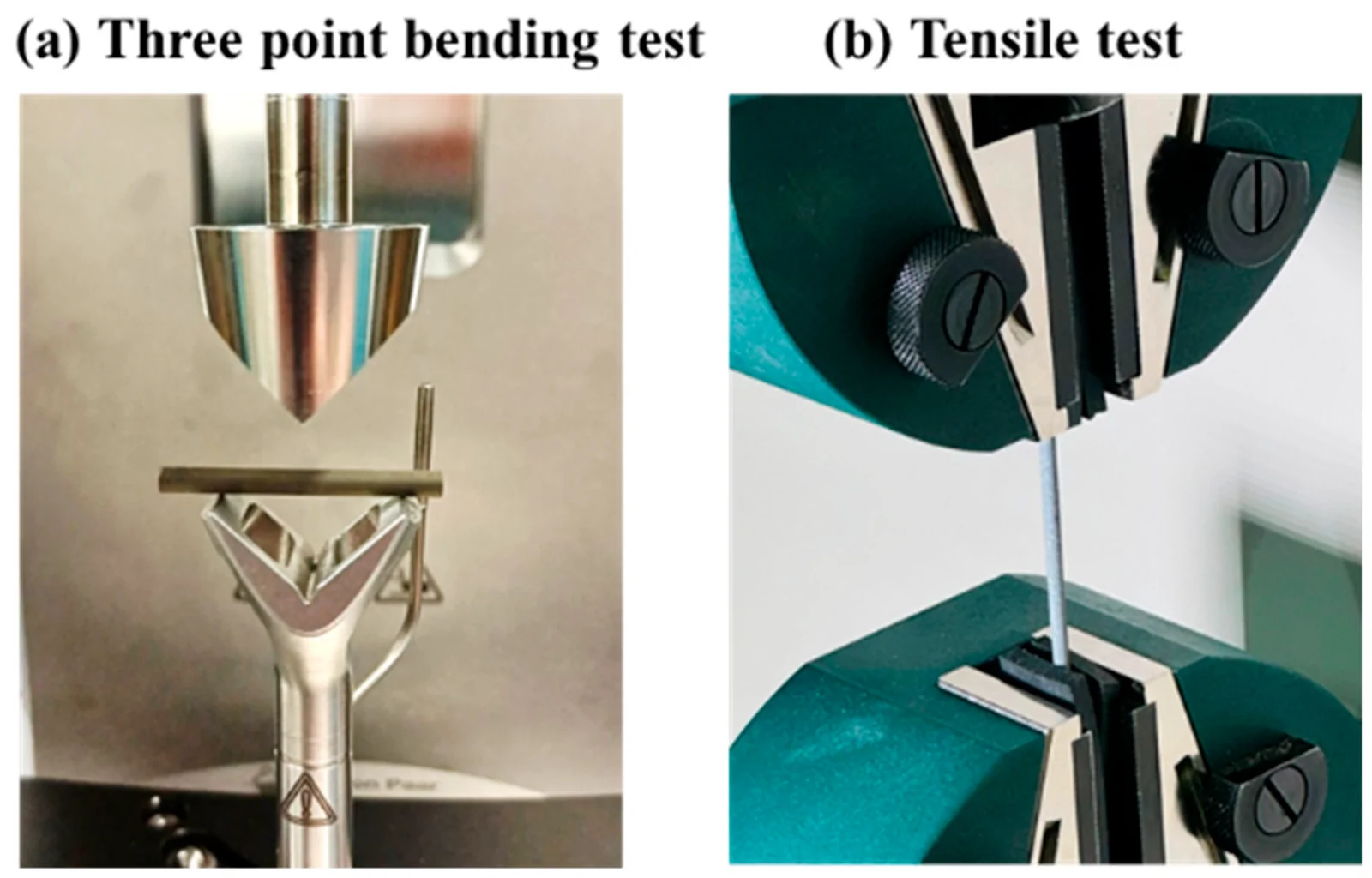
Experimental setups used for mechanical testing of feedstock filaments: (**a**) three-point bending test and (**b**) tensile test.

Three-point bending tests were performed on filament specimens with a length of 30 mm using a rheological testing system (MCR702, Anton Paar, Graz, Austria) equipped with a three-point bending fixture, as shown in Figure 4a. The support span was 20 mm, and the force was increased at a rate of 1 N s^−1^ up to a maximum force of 40 N. The maximum flexural stress, flexural strain, and flexural modulus were determined from the load–deflection curves. The maximum flexural stress was calculated according to Equation (1):

(1) $$\sigma_{max}=\frac{FL}{\pi R^{3}}$$

where σ_max_ is the maximum flexural stress, F is the applied force, L is the support span, and R is the filament radius. The flexural modulus, E_f_, was calculated using Equation (2):

(2) $$E_{f}=\frac{L^{3}m}{4\pi R^{4}}$$

where *m* is the slope of the linear region of the load–deflection curve.

Tensile tests were performed on straight filament specimens with a length of 100 mm using a universal testing machine equipped with a 1 kN load cell, as shown in Figure 4b. The gauge length was 50 mm. The filaments were clamped using wedge grips and tested at a crosshead speed of 10 mm min^−1^ until rupture. The deformation was analyzed using Istra4D software (version 4.6).

### 2.8. MEX-Printed Ring Specimens

The printability of the developed feedstock filaments, F1_Sm and F2_Sr, was evaluated using a MEX printer (HAGE3D, Graz, Austria). Ring-shaped specimens with an outer diameter of 33 mm, an inner diameter of 26 mm, and a height of 4.3 mm were fabricated. The models were sliced, and G-code was generated using Simplify3D software (version 4.1.2, Simplify3D, Blue Ash, OH, USA). Printing was performed using a brass HAGE3D nozzle with a diameter of 0.8 mm. The same printing parameters were used for both feedstocks to evaluate their processability within a common MEX processing window.

The build plate consisted of a glass substrate covered with commercially available adhesion tape. The tape surface was mechanically roughened using P500-grade sandpaper to improve first-layer adhesion. Similar printing parameters were used to fabricate the ring-shaped specimens from both feedstocks.

### 2.9. Bulk Density Measurement

The bulk density of the extruded filaments and MEX-printed specimens was determined using a Densitec bulk-density meter (Exelia AG, Zürich, Switzerland). The measurement was based on Archimedes’ principle using low-viscosity silicone oil as the immersion medium. Before measurement, the specimens were dried at 40 °C for 2 h, weighed in air, and then fully submerged in silicone oil with a density of 0.965 g cm^−3^ at 23 °C. The bulk density was calculated from the measured mass and buoyancy response. Three independent measurements were performed for each sample, and the relative standard deviation was below 2%.

### 2.10. X-Ray Computed Microtomography (Micro-CT)

X-ray computed microtomography (micro-CT) was used to characterize the internal morphology of the extruded filaments and MEX-printed ring specimens. Measurements were performed using an EasyTom XL 160 Ultra system (RX Solutions, Chavanod, France) equipped with a nanofocus X-ray source operated in the range of 20–160 kVp, with a maximum power of 32 W, and a 16-bit flat-panel detector.

The ring specimens, with an outer diameter of approximately 33 mm, were fixed to the sample holder and scanned along the rotation axis. The scans were acquired at 140 kVp and approximately 47–48 µA using a 1.5 mm Al filter. A total of 2400 projections were collected over a 360° continuous rotation at 1 fps, with four frames averaged per projection. The effective pixel size was approximately 14 µm.

Both filament types, with a diameter of approximately 2.85 mm, were mounted together with their long axes perpendicular to the rotation stage. A filament section of approximately 11 mm was scanned at 120 kVp and 77 µA. For these scans, 2400 projections were collected over a 360° continuous rotation at 1 fps, with six frames averaged per projection. The effective pixel size was approximately 5.5 µm.

Projection data were reconstructed into axial 16-bit TIFF image stacks using X-ACT software (https://www.rx-solutions.com/en/products/x-act-tomography-software-1281 accessed on 26 February 2026) CT scanning suite (RX Solutions, Chavanod, France). The reconstructed image stacks were used to assess internal defects, porosity, and filament/ring structural homogeneity. Two-dimensional visualization was performed using ImageJ 1.51p, and three-dimensional renderings were generated using Dragonfly software (2024.1, Comet Technologies, Montreal, QC, Canada).

## 3. Results

### 3.1. Thermal Properties of Filaments

**Figure 5:**
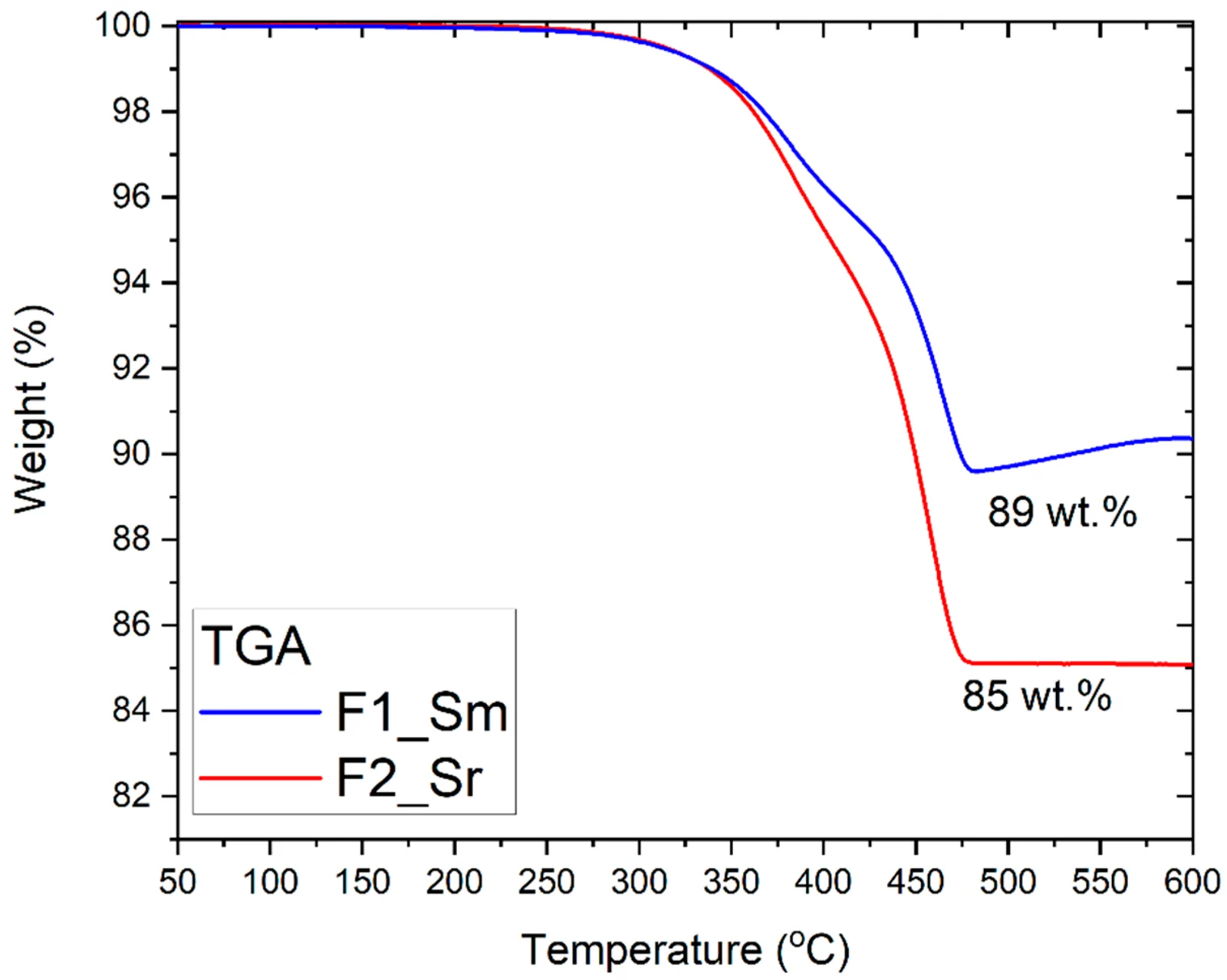
TGA curves of the extruded magnetic filaments F1_Sm and F2_Sr measured under nitrogen atmosphere.

The thermal stability and residual powder content of the extruded magnetic filaments were evaluated by TGA, as shown in Figure 5. Both filaments remained thermally stable up to approximately 330 °C, with only minor mass loss observed in this temperature range. The main mass loss occurred between approximately 350 and 500 °C and is attributed to thermal decomposition of the polymer binder system. No additional thermal transition was detected by DSC up to 300 °C, apart from the weak glass transition and the melting transition discussed below. After binder decomposition, the residual mass corresponded to the magnetic powder fraction in the filaments.

The residual mass was approximately 89 wt.% for F1_Sm and 85 wt.% for F2_Sr. These values are close to the targeted high powder loading of 85–90 wt.%, confirming that highly filled feedstock filaments were obtained after compounding and extrusion. The measured bulk densities were 4.25 g cm^−3^ for F1_Sm and 3.10 g cm^−3^ for F2_Sr (Table 3). Based on the measured densities and powder densities, these values correspond to estimated magnetic solid contents of approximately 52 vol.% for F1_Sm and 51 vol.% for F2_Sr. Therefore, both feedstocks reached powder contents suitable for highly filled MEX filaments.

**Figure 6:**
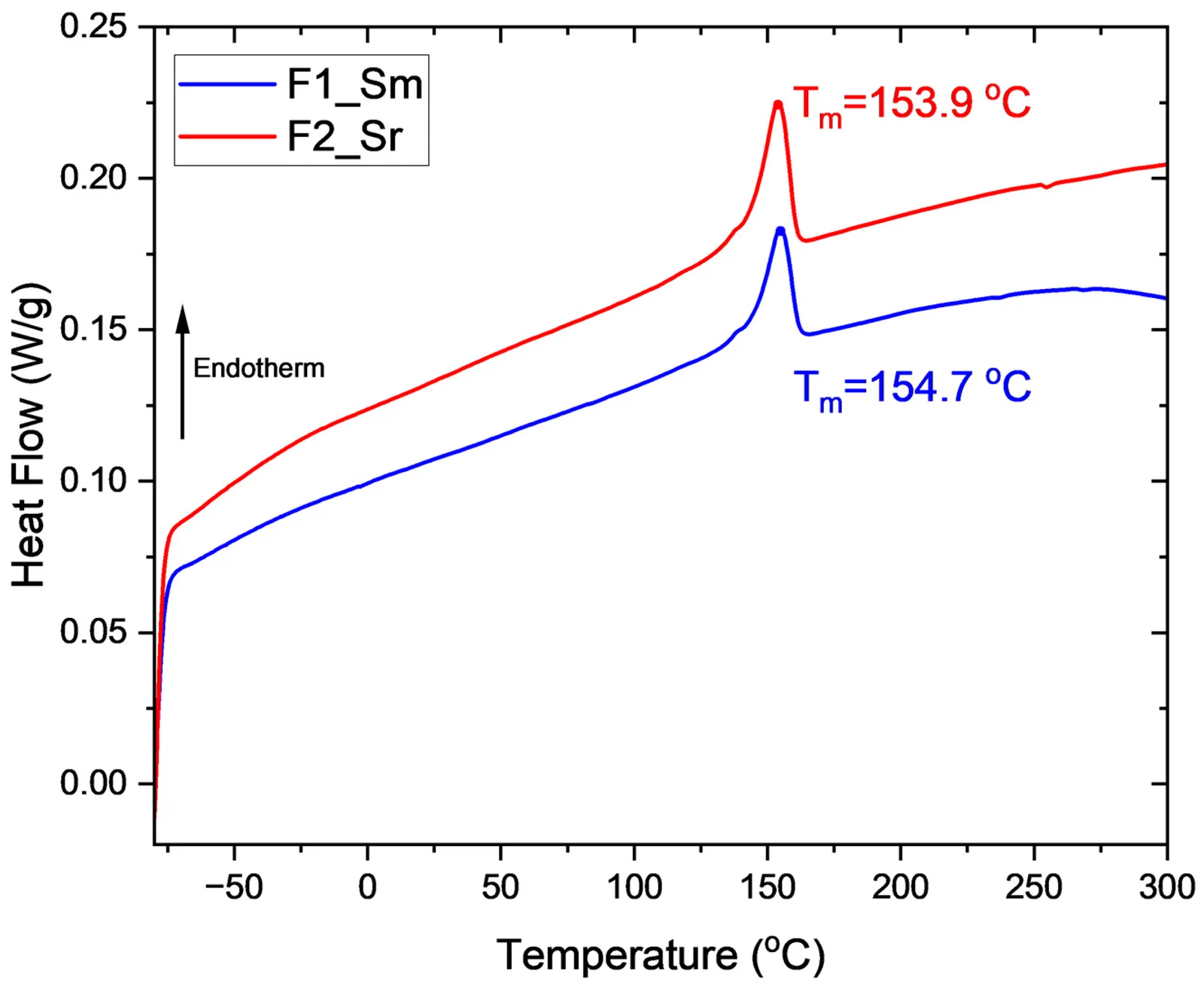
DSC first-heating thermograms of F1_Sm and F2_Sr composite filaments measured from −80 to 300 °C at 10 K min^−1^ under nitrogen. Heat flow is normalized to sample mass; endothermic direction is upward.

The DSC first-heating thermograms of the composite filaments are shown in Figure 6. Both F1_Sm and F2_Sr exhibited a clear melting transition between approximately 100 and 170 °C. The melting peak temperatures were very similar, with T_m_ values of 154.7 °C for F1_Sm and 153.9 °C for F2_Sr. The melting enthalpy was slightly higher for F2_Sr than for F1_Sm, increasing from 4.3 to 4.9 J g^−1^. This difference is consistent with the lower inorganic filler content of F2_Sr, which corresponds to a higher relative polymer fraction.

A very weak glass transition was detected in the DSC data, but it was difficult to determine accurately because of the low polymer fraction and the weak transition signal in these highly filled composites. The estimated T_g_ values were approximately −52 °C for F1_Sm and −49 °C for F2_Sr. This small difference is not considered significant within the uncertainty of the measurement. Apart from the weak glass transition and the melting transition, no additional thermal transition was detected by DSC up to 300 °C.

### 3.2. Morphology of Filaments

**Figure 7:**
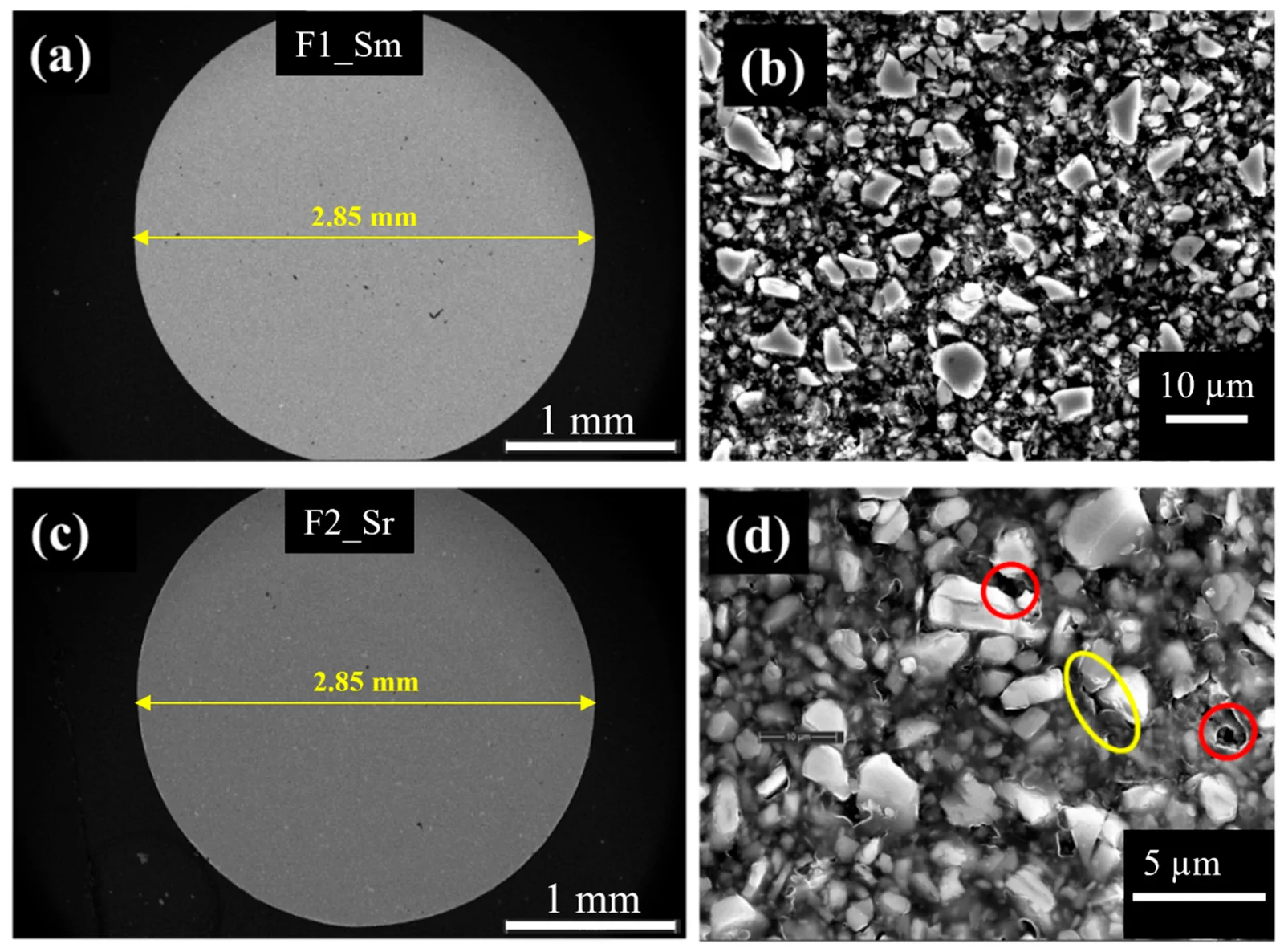
Cross-sectional SEM images of extruded feedstock filaments: (**a**,**b**) F1_Sm and (**c**,**d**) F2_Sr. In (**d**), the yellow circle indicates local particle–binder separation, and the red circles indicate microvoids.

Cross-sectional SEM images of the extruded filaments are shown in Figure 7a–d. At low magnification, both F1_Sm and F2_Sr exhibited dense, highly filled cross-sections without visible macroscopic defects. Both filament cross-sections appeared nearly circular and showed uniform diameters (2.85 mm), indicating stable filament formation during extrusion. At higher magnification, however, differences in particle distribution and particle–binder interaction were observed. The F1_Sm filament showed a comparatively uniform SmFeN particle network, with good binder coverage and limited interfacial gaps between particles and the polymer matrix. This morphology suggests effective powder dispersion and binder wetting during compounding and extrusion.

In contrast, the F2_Sr filament showed generally good particle dispersion but less homogeneous binder coverage. Localized microvoids and regions of particle–binder separation were observed, as indicated in Figure 7d. These features may act as local stress concentrators and can influence the mechanical response of the filament, particularly stiffness and deformation behavior.

### 3.3. CT Measurement on Filaments

**Figure 8:**
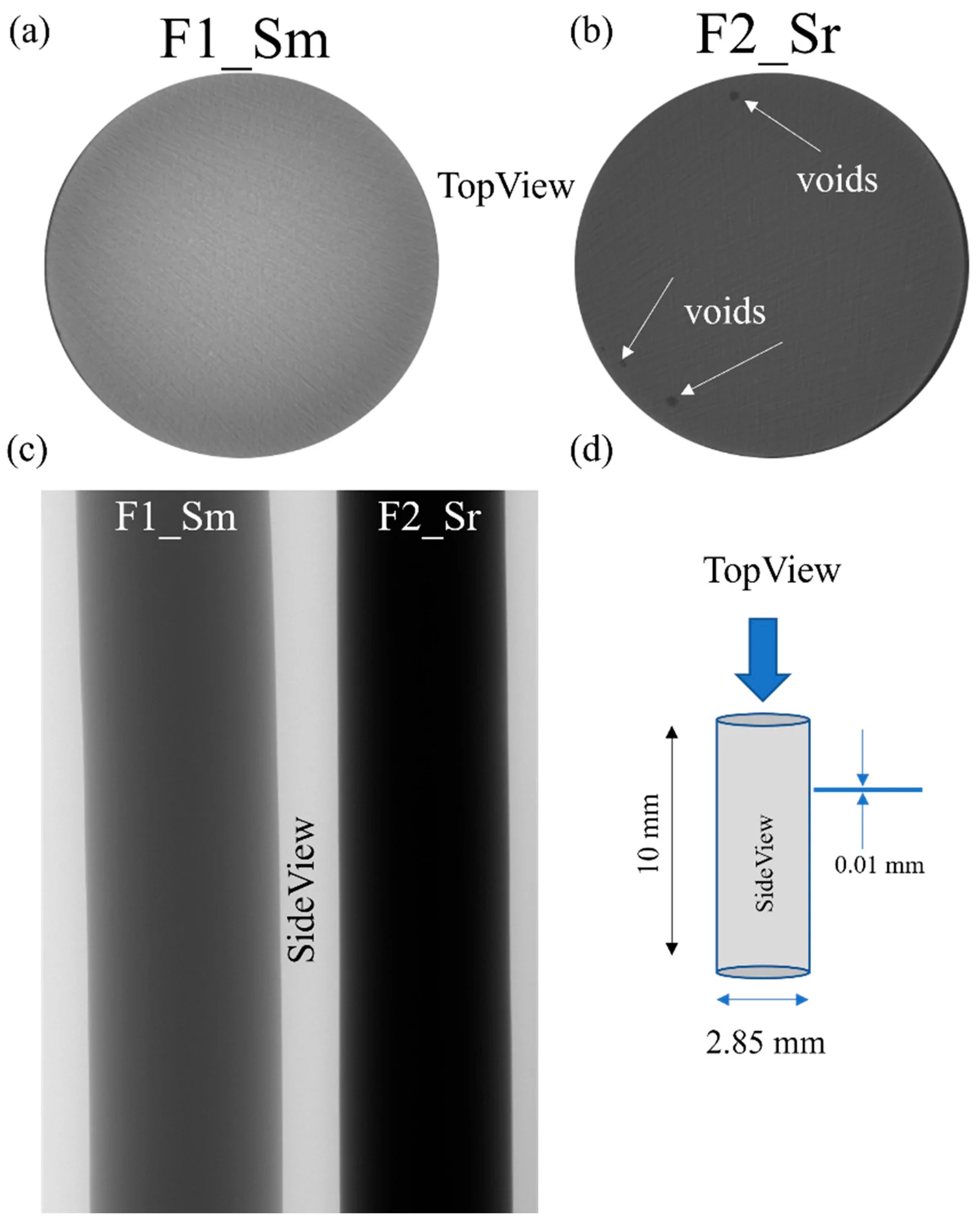
Micro-CT reconstructions of the extruded filaments: (**a**) top view of the F1_Sm showing a comparatively uniform cross-section, (**b**) top view of the F2_Sr showing localized internal voids, (**c**) side-view comparison of the F1_Sm and F2_Sr filaments, and (**d**) schematic representation of the top-view and side-view orientations.

Micro-CT analysis was used to evaluate the internal structure of the extruded filaments, as shown in Figure 8a–d. The reconstructed top-view images show clear differences between the two filament types. The SFO-based filament (F2_Sr) contains discrete low-attenuation regions, visible as dark spots, which are consistent with internal voids or pores. These features appear localized within the filament cross-section rather than uniformly distributed. In contrast, the SmFeN-based filament (F1_Sm) shows a more homogeneous grayscale contrast, with no large voids visible under the same visualization conditions.

The side-view reconstructions further support these observations. The SFO filament exhibits stronger contrast variations along the filament length, suggesting local density variations and/or internal porosity. The SmFeN filament shows a more uniform attenuation profile along the extrusion direction, indicating more homogeneous packing and structural continuity. The pores observed in the SFO filament were approximately circular and had characteristic sizes of about 30–40 µm. These micro-CT observations are consistent with the SEM results, where localized voids and particle–binder separation were also observed in the SFO-based filament.

The higher structural homogeneity of the SmFeN filament suggests more uniform powder distribution and binder wetting during compounding and extrusion. In contrast, the localized porosity in the SFO filament may reduce the effective load-bearing area and act as stress-concentration sites during mechanical testing. These features are therefore expected to influence filament stiffness, strength, and feeding reliability during MEX processing.

### 3.4. Mechanical Testing of Feedstock Filaments

**Figure 9:**
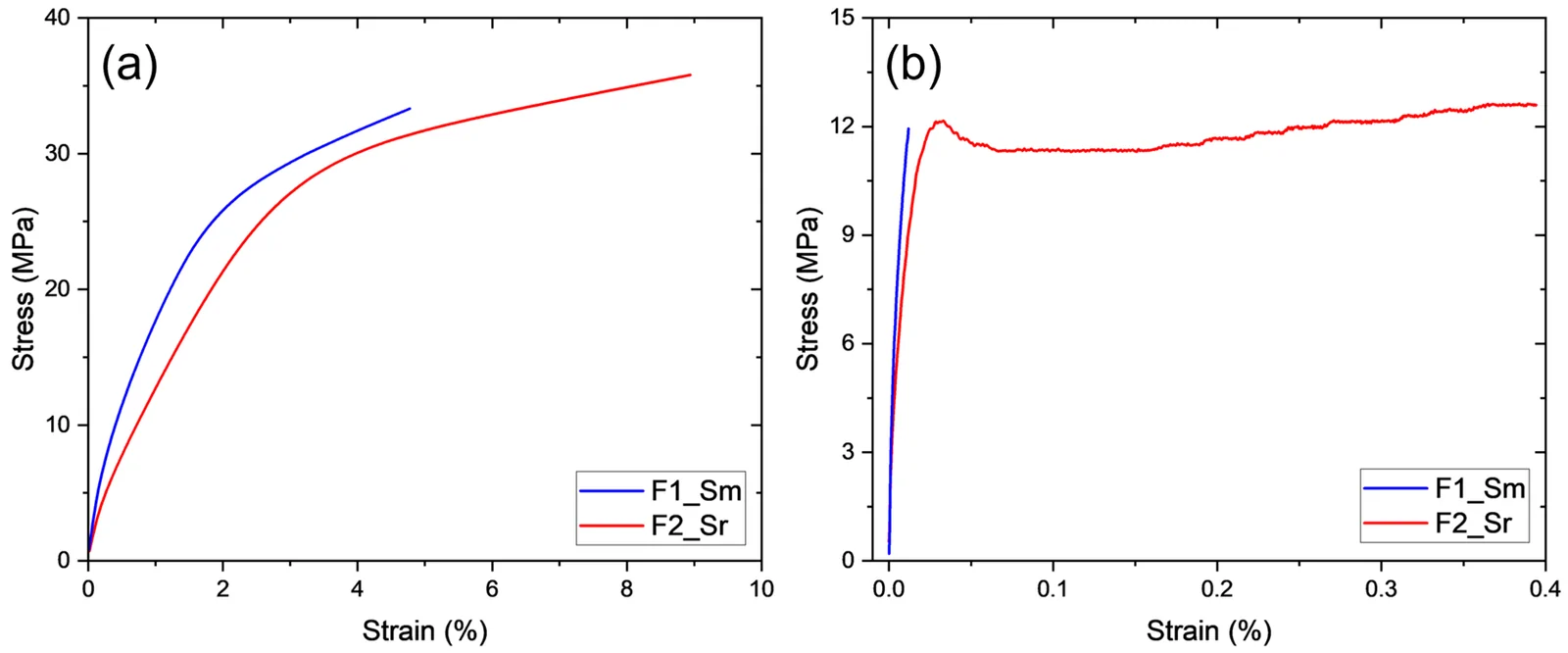
Stress–strain curves of the extruded feedstock filaments F1_Sm and F2_Sr: (**a**) three-point bending test using a 40 N load cell and (**b**) tensile test using a 1 kN load cell.

Three-point bending and tensile tests were performed to evaluate the mechanical suitability of the extruded feedstock filaments for handling, spooling, and MEX feeding. The representative stress–strain curves are shown in Figure 9a,b, and the calculated mechanical properties are summarized in Table 4.

In three-point bending, both filaments showed continuous deformation without fracture during the test, indicating sufficient flexibility for handling and automatic spooling. The SFO-based filament, F2_Sr, exhibited the higher maximum flexural stress of 36.12 ± 0.14 MPa, while F1_Sm showed a value of 31.45 ± 1.77 MPa. In contrast, the SmFeN-based filament, F1_Sm, showed the higher flexural modulus of 5.5 ± 0.2 GPa, compared with 3.0 ± 0.04 GPa for F2_Sr. The ± values represent the standard deviation of replicate measurements. This indicates that F1_Sm was stiffer, whereas F2_Sr tolerated higher flexural stress under the applied test conditions.

The tensile results showed comparable trends. F2_Sr exhibited a slightly higher ultimate tensile strength (UTS) of 12.28 ± 0.39 MPa, while F1_Sm showed a UTS of 11.40 ± 0.73 MPa. However, F1_Sm again showed the higher Young’s modulus of 2.1 ± 0.01 GPa, compared with 1.7 ± 0.03 GPa for F2_Sr. These values are within the range reported for highly filled feedstock filaments and are suitable for filament handling and feeding during MEX processing [14,16,18,19].

**Figure 10:**
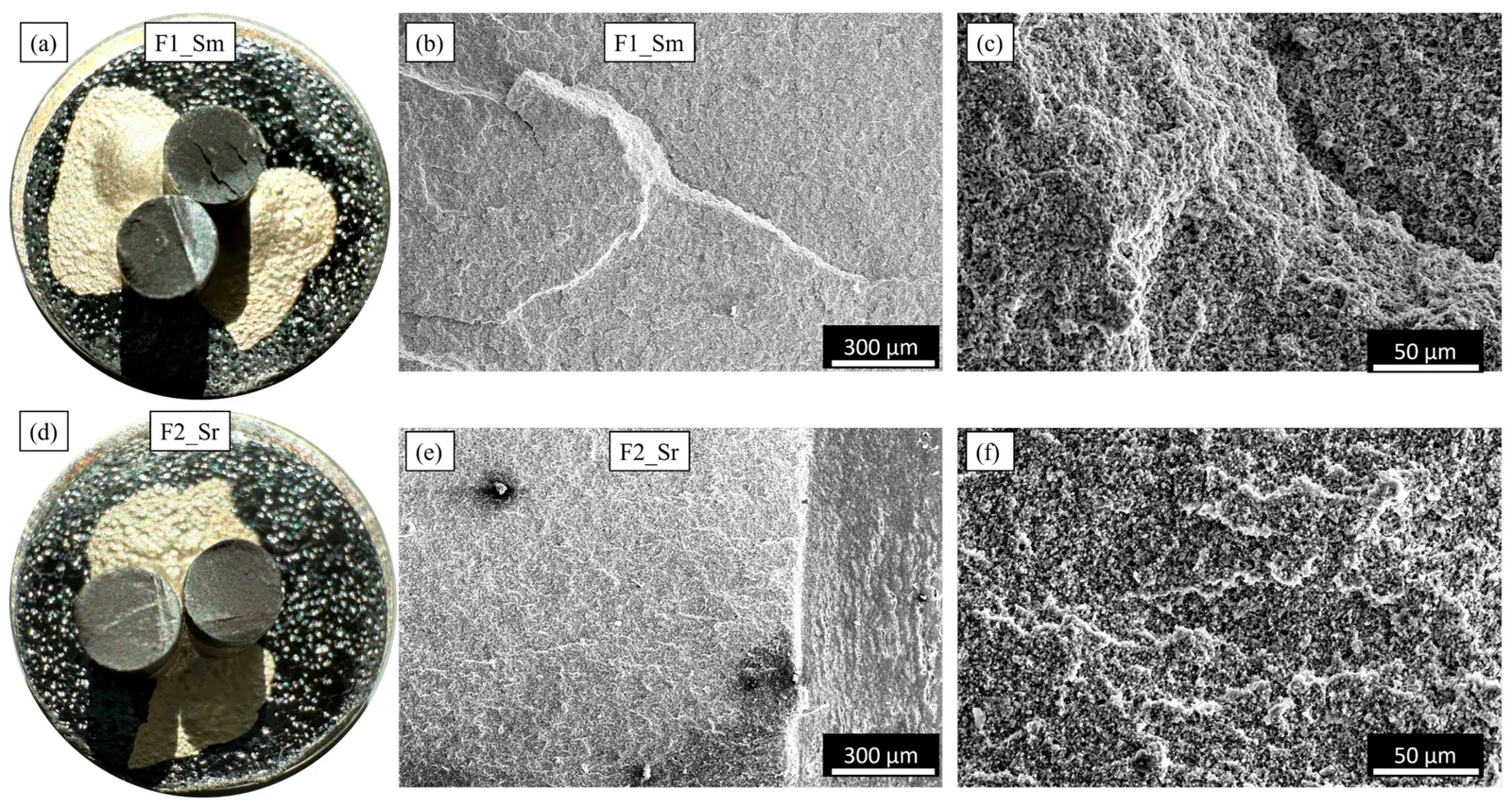
Optical and SEM images of fractured tensile specimens of (**a**–**c**) F1_Sm and (**d**–**f**) F2_Sr after tensile testing.

Optical and SEM observations of the fractured tensile specimens were used to clarify the failure mode, as shown in Figure 10a–f. Both F1_Sm and F2_Sr exhibit relatively flat macroscopic fracture surfaces, indicating brittle-dominated failure under tensile loading. The F1_Sm specimen shows more visible secondary cracking in the optical image, while SEM analysis reveals rough fracture ridges and an irregular crack path through the composite microstructure. In F2_Sr, high-magnification SEM images show a particle-rich fracture surface with numerous exposed filler particles and local void-like features, suggesting localized filler–matrix debonding or particle pull-out during crack propagation. However, no evidence of extensive, continuous magnetic filler delamination is observed in either material. Therefore, the tensile failure is interpreted as brittle fracture through the polymer-bonded composite microstructure, accompanied by localized interfacial debonding and particle exposure rather than large-scale filler delamination.

### 3.5. Rheological Properties of Feedstocks

**Figure 11:**
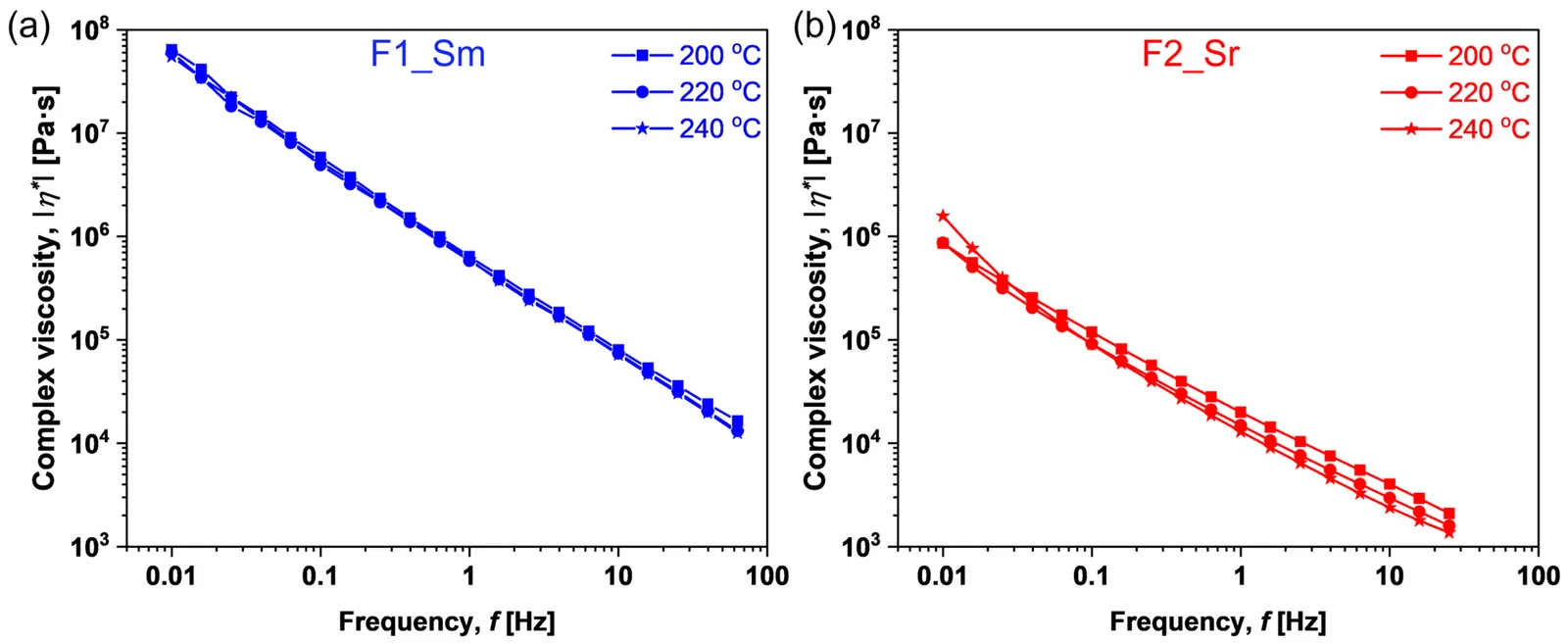
Complex viscosity, η*, as a function of frequency for magnetic feedstocks at 200, 220, and 240 °C: (**a**) F1_Sm and (**b**) F2_Sr.

Rheological analysis was performed to evaluate the melt flow behavior and viscoelastic response of the highly filled feedstocks during MEX processing. Figure 11 shows the complex viscosity, η*, as a function of frequency at 200, 220, and 240 °C. Both F1_Sm and F2_Sr exhibited pronounced shear-thinning behavior over the investigated frequency range, with η* decreasing continuously as frequency increased. This behavior is favorable for MEX because the feedstock can flow more easily under the high-shear conditions inside the nozzle, while higher viscosity at lower shear rates can support strand stability after deposition [16].

F1_Sm showed little temperature dependence, with nearly overlapping viscosity curves at 200, 220, and 240 °C. In contrast, F2_Sr showed a clearer decrease in viscosity with increasing temperature, especially the change in slope at low frequencies. This difference suggests that the stronger particle–binder network in F1_Sm limited the effect of binder softening, whereas the weaker internal network in F2_Sr relaxed more easily at higher temperatures.

**Figure 12:**
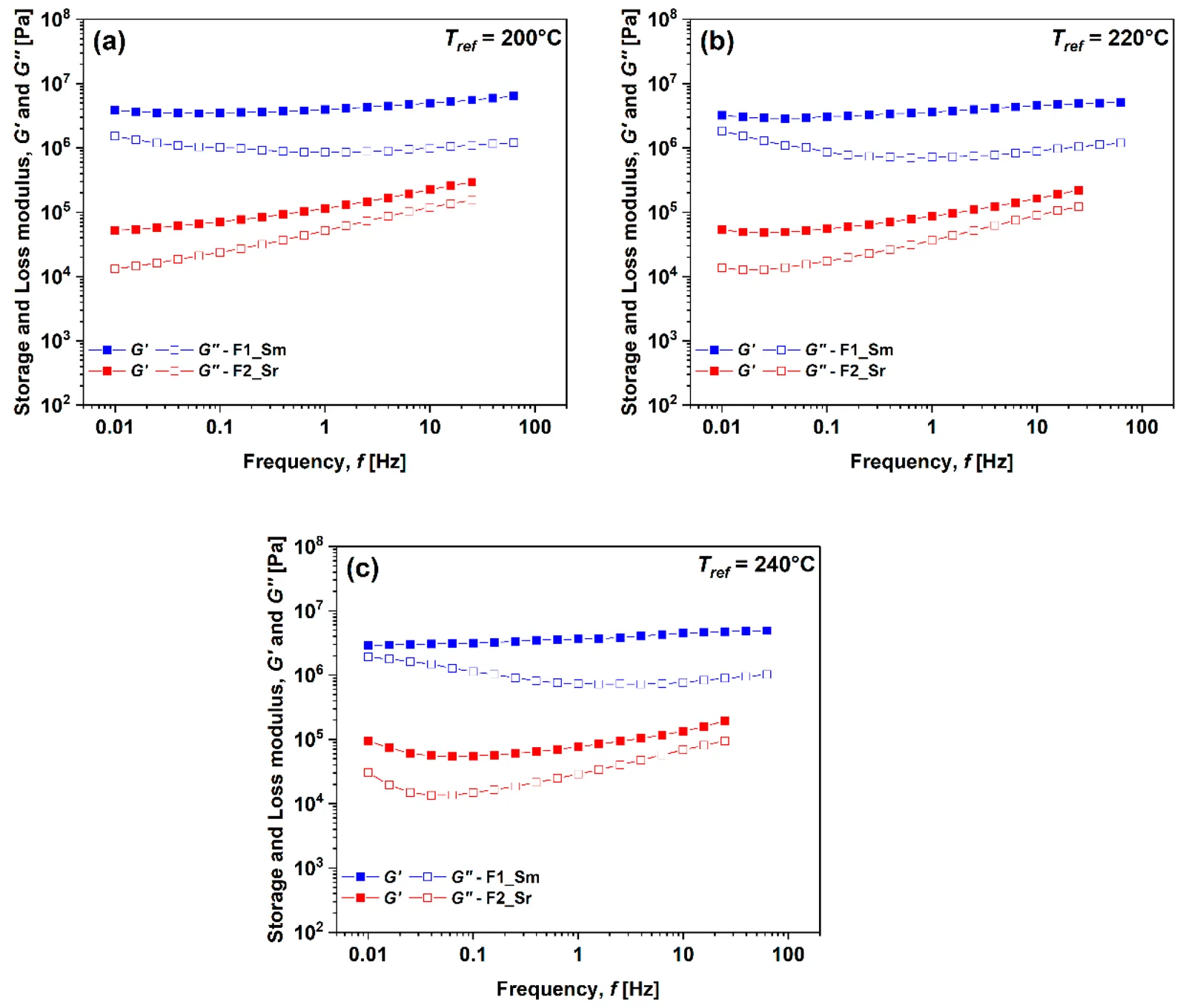
Storage modulus, G′, and loss modulus, G″, as a function of frequency for F1_Sm and F2_Sr at (**a**) 200, (**b**) 220, and (**c**) 240 °C.

The viscoelastic properties are shown in Figure 12a–c. For both feedstocks and at all tested temperatures, the storage modulus, G′, was higher than the loss modulus, G″, across the investigated frequency range. This elastic-dominant response indicates the presence of a particle-supported network within the highly filled feedstocks. Such behavior is beneficial for MEX, as it helps the extruded strand retain its shape after deposition. The absolute values of G′ and G″ followed the same material-dependent trend as the complex viscosity, with F1_Sm showing higher moduli than F2_Sr. For F1_Sm, the moduli showed only limited temperature dependence, suggesting that the stronger particle-supported network dominated the response. In contrast, F2_Sr showed clearer temperature-dependent changes, especially at low frequencies, which is consistent with partial relaxation of a weaker particle-supported network, reduced binder viscosity, and local particle rearrangement in binder-rich regions. Overall, both feedstocks exhibited shear-thinning behavior and elastic-dominant viscoelasticity, indicating a suitable rheological processing window for MEX printing.

### 3.6. Magnetic Properties of Feedstock Filaments

**Figure 13:**
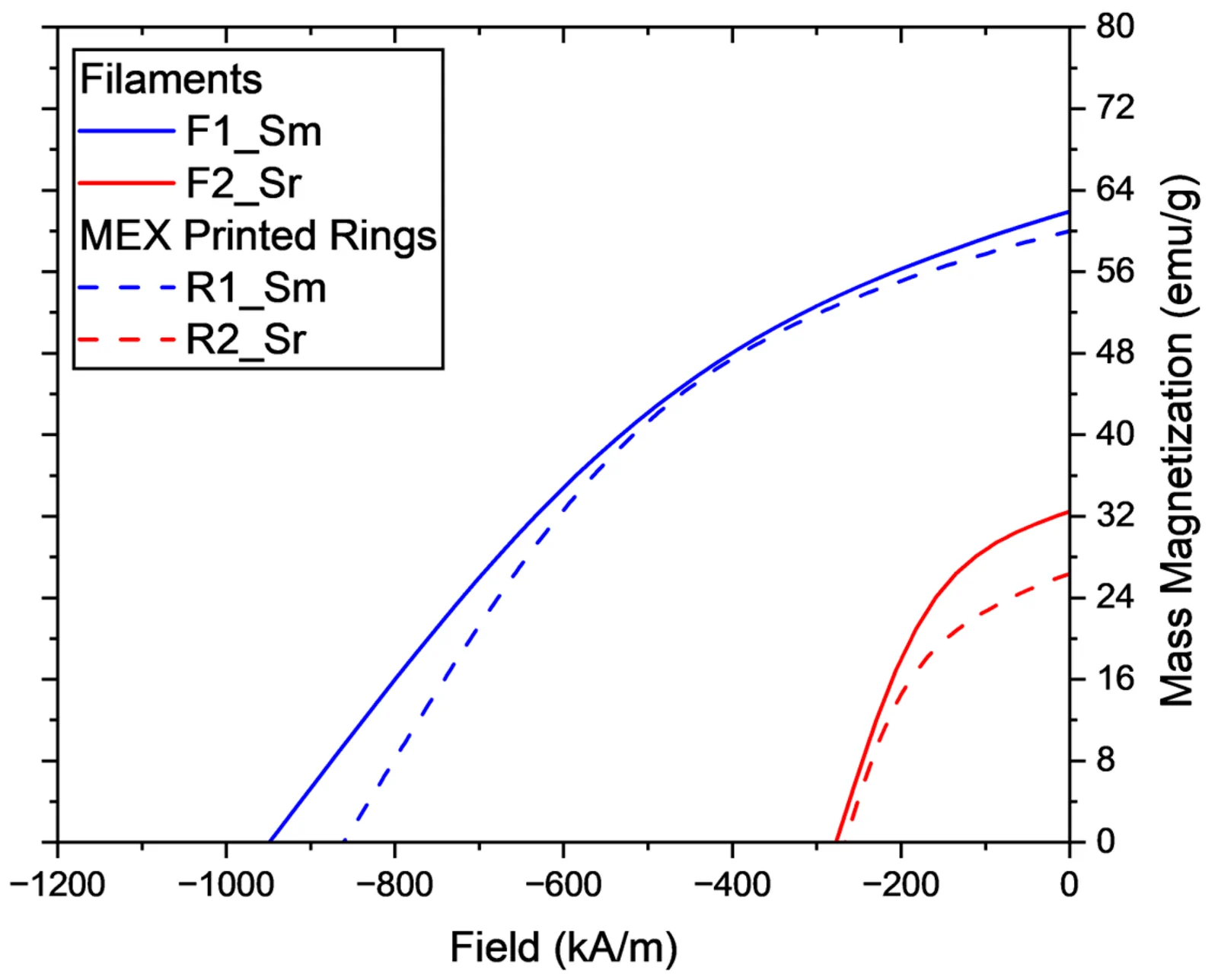
Second-quadrant VSM demagnetization curves of the extruded feedstock filaments (F1_Sm and F2_Sr) and MEX printed rings (R1_Sm and R2_Sr). Magnetization is reported as mass magnetization, normalized to the total sample mass.

The magnetic properties of the extruded filaments are shown in Figure 13 (solid lines) and were compared with those of the corresponding starting powders. After compounding and filament extrusion, both feedstocks showed lower mass-normalized M_r_ than the starting powders. For F1_Sm, M_r_ decreased from approximately 78 emu/g for the powder to 62 emu/g for the filament, while H_c_ decreased from 1110 to 950 kA/m. For F2_Sr, M_r_ decreased from approximately 36 to 32 emu/g, while H_c_ decreased from 296 to 278 kA/m.

The reduction in M_r_ is mainly attributed to dilution by the non-magnetic polymer binder, because the VSM signal was normalized to the total mass of the composite filament rather than only to the magnetic powder mass. Therefore, the lower magnetic powder fraction in the filament directly reduces the measured mass magnetization. In contrast, H_c_ is not expected to decrease solely due to polymer dilution, since coercivity is primarily governed by the magnetic powder characteristics, including particle size, morphology, surface condition, and domain reversal behavior. The moderate reduction in H_c_ after processing may therefore be related to changes introduced during compounding and extrusion, such as particle surface modification, minor oxidation, local damage, altered particle–particle interactions, or differences in demagnetizing effects between powders and composite filaments. Overall, the VSM results indicate that the hard-magnetic character of both fillers was largely retained after filament fabrication, with the main change being the expected decrease in mass-normalized M_r_ due to the binder fraction.

### 3.7. Additive Manufacturing by MEX

**Figure 14:**
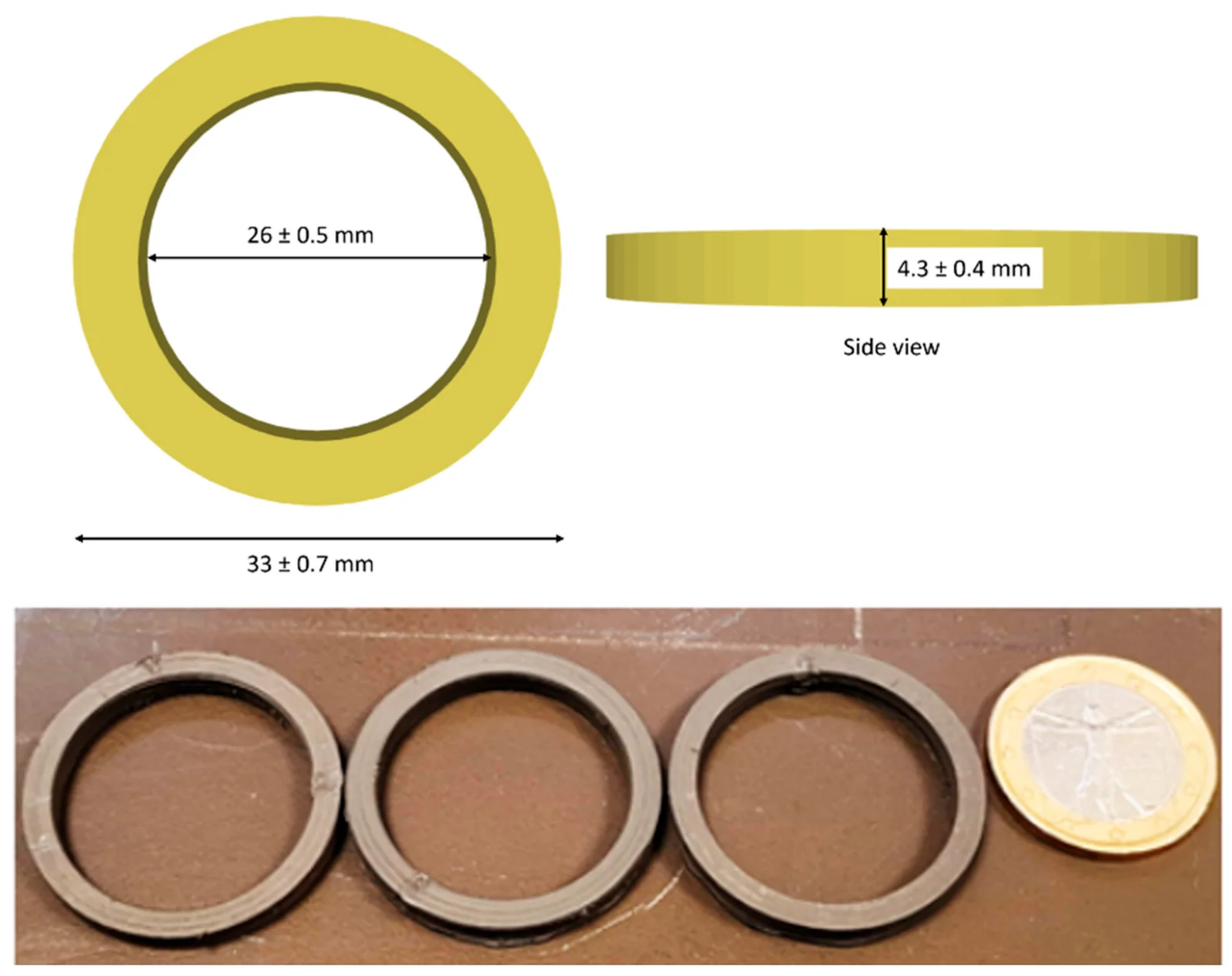
Geometry and photographs of MEX-printed ring specimens. (**Top**): schematic of the ring geometry with nominal dimensions. (**Bottom**): as-printed ring specimens produced from SmFeN-based filament (F1_Sm) and SFO-based filament (F2_Sr); coin shown for scale.

The printability of the highly filled feedstock filaments was evaluated by fabricating ring-shaped specimens using MEX. Both F1_Sm and F2_Sr were successfully printed using the same nozzle diameter and a common set of printing parameters, summarized in Table 5. The printed specimens, denoted as R1_Sm and R2_Sr, are shown in Figure 14. The ability to process both feedstocks under the same printing window indicates that the TPE + gPO binder system provided sufficient melt flow through the nozzle and adequate strand stability after deposition.

The selected printing parameters were guided by the rheological behavior discussed above. Both feedstocks exhibited shear-thinning behavior, which supports flow under the high-shear conditions inside the nozzle, and elastic-dominant behavior (G′ > G″), which helps the deposited strand retain its shape. The nozzle temperature of 220–230 °C was selected to provide stable extrusion while avoiding excessive softening of the deposited material. A 0.8 mm nozzle was used to reduce the risk of clogging and to support the flow of highly filled feedstocks.

Good first-layer adhesion was achieved using roughened adhesive tape on a glass build plate. The chamber temperature was maintained at 55–60 °C to reduce thermal gradients during printing and support interlayer bonding. This moderate chamber temperature helped maintain dimensional stability while promoting sufficient contact and healing between adjacent deposited strands. Overall, the successful fabrication of dimensionally consistent ring specimens confirms that both highly filled magnetic filaments can be processed by MEX within a shared processing window.

### 3.8. Characterization of MEX-Printed Rings

**Figure 15:**
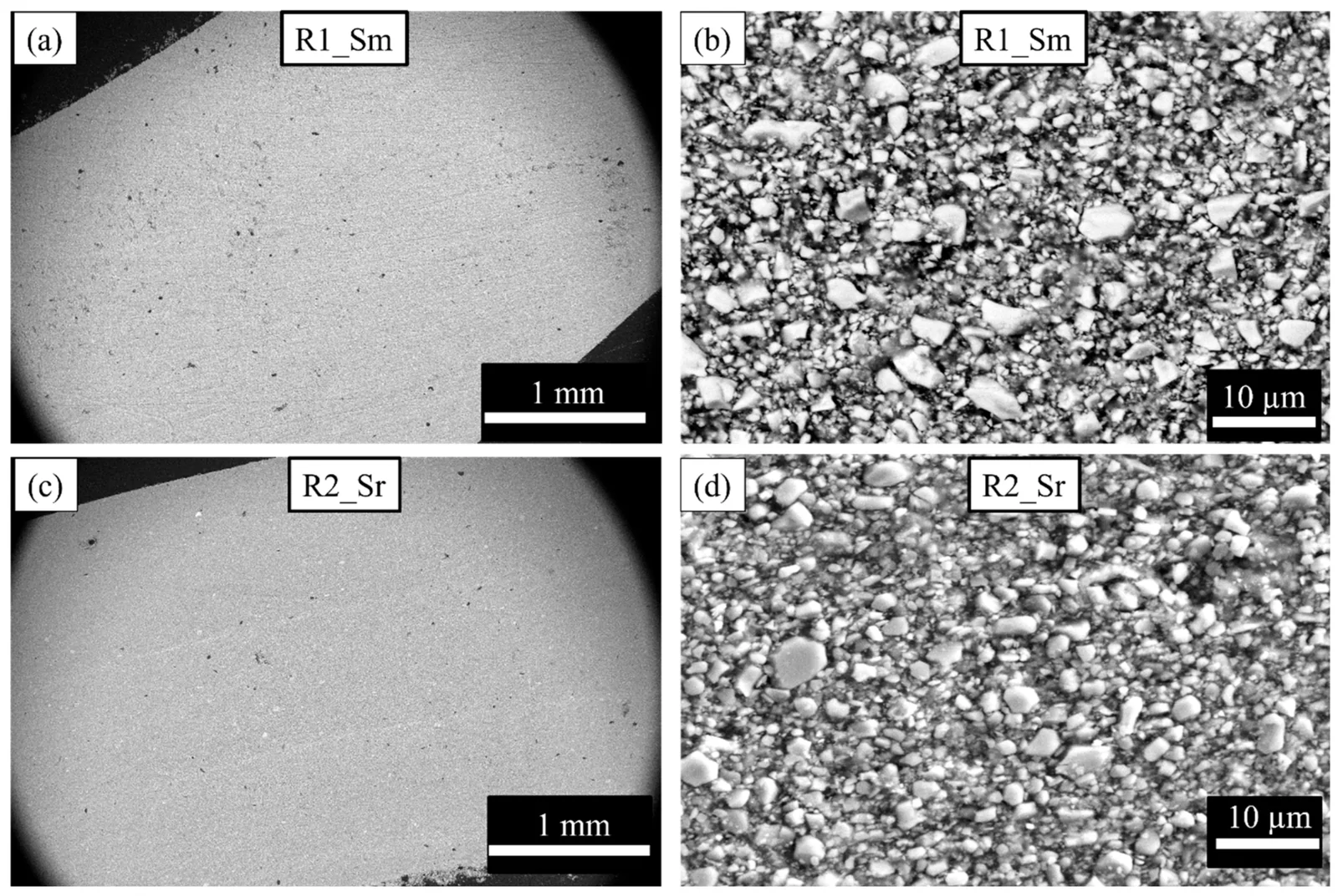
SEM micrographs of MEX-printed ring specimens: (**a**,**b**) R1_Sm and (**c**,**d**) R2_Sr.

The morphology of the MEX-printed ring specimens was examined by SEM, as shown in Figure 15. At low magnification, both R1_Sm and R2_Sr showed dense cross-sections with no large macroscopic defects. The raster structure was only weakly visible, indicating good consolidation between deposited strands. At higher magnification, R1_Sm showed a comparatively uniform particle–binder network, with good particle dispersion and limited interfacial gaps. R2_Sr also showed a highly filled structure, although the particle–binder distribution appeared slightly less homogeneous, consistent with the morphology observed in the corresponding filament.

**Figure 16:**
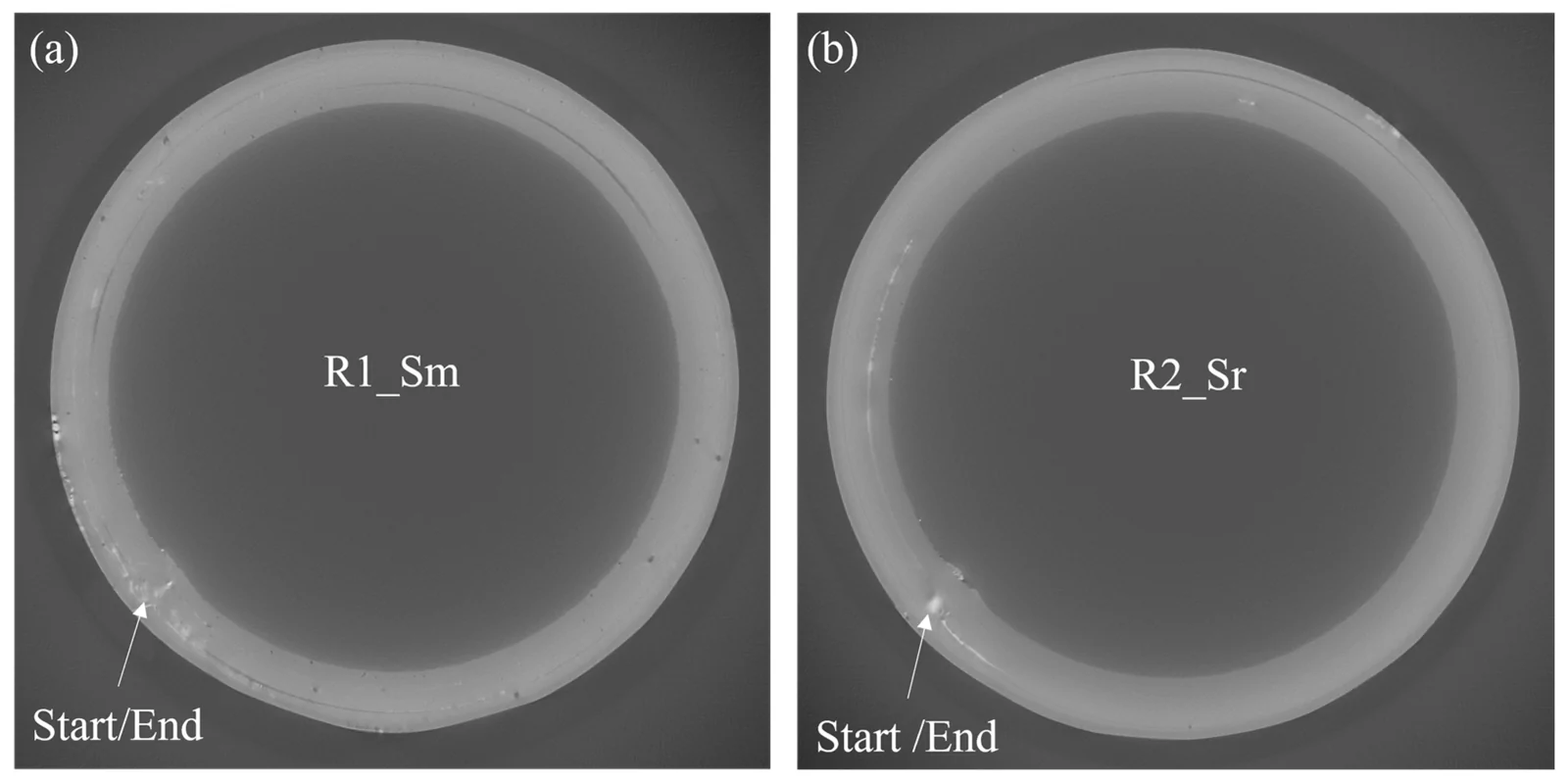
Micro-CT reconstructions of MEX-printed ring-shaped magnets: (**a**) R1_Sm and (**b**) R2_Sr. The arrows indicate the deposition start/end region, where local material accumulation is observed.

Micro-CT was further used to evaluate the internal structure of the printed rings. The reconstructed images, shown in Figure 16, showed continuous ring geometries for both SFO- and SmFeN-based specimens, with no large internal voids or major structural discontinuities visible at the scanned resolution. Minor local contrast variations were observed near the outer regions of the rings, which may be related to surface roughness, local bead overlap, or small density variations introduced during layer deposition. The arrows in Figure 16 indicate the start/end region of ring deposition, where the lower print-head velocity during path initiation and termination can locally increase material residence and promote additional particle deposition. Overall, the CT results confirm that both feedstocks could be printed into structurally continuous ring specimens using the selected MEX processing window.

The magnetic properties of the printed rings were measured with VSM using small representative sections cut from the rings, as the complete ring geometry could not be mounted in the VSM sample holder. The results are shown in Figure 13 (dotted lines) and summarized in Table 6. The printed rings retained the hard-magnetic character of the corresponding filaments. For the SmFeN-based material, M_r_ decreased slightly from 62 emu/g for F1_Sm to 60 emu/g for R1_Sm, while H_c_ decreased from 950 to 863 kA/m. For the SFO-based material, M_r_ decreased from 32 emu/g for F2_Sr to 26 emu/g for R2_Sr, while H_c_ decreased from 278 to 264 kA/m.

The small reduction in mass-normalized M_r_ after printing may be associated with processing-related effects such as local density variations, strand deposition history, and possible changes in particle packing during nozzle extrusion. The decrease in H_c_ was moderate for both materials, indicating that the hard-magnetic behavior was largely preserved after MEX printing. Overall, the combined SEM, micro-CT, and VSM results show that the selected binder system enabled the fabrication of dense, structurally continuous, and magnetically functional MEX-printed ring specimens.

## 4. Discussion

The results show that powder dispersion, particle–binder interaction, and internal defects strongly influence the mechanical, rheological, and printing behavior of highly filled magnetic filaments for MEX. This agrees with previous work on highly filled extrusion feedstocks, where powder characteristics, binder composition, and powder-binder compatibility were shown to control flow behavior, filament integrity, and printability [13,14,16,19,20,21]. In the present study, the TPE + gPO binder system provided a common processing window for both SmFeN- and SFO-based feedstocks, although the two powders produced different microstructural and flow responses. SEM and micro-CT observations showed that the SmFeN-based filament formed a more homogeneous particle–binder network, whereas the SFO-based filament contained localized voids and less uniform binder coverage. These differences were reflected in the mechanical properties: F1_Sm showed higher flexural and tensile modulus, while F2_Sr showed slightly higher maximum flexural stress and UTS. This confirms that filler morphology, packing, and interfacial quality play a central role in determining the performance of highly filled feedstock filaments.

The rheological results further support this interpretation. Both feedstocks exhibited pronounced shear-thinning behavior, which is beneficial for MEX because it supports flow through the nozzle under high shear while allowing the deposited strand to retain its shape after extrusion. At the same time, the storage modulus exceeded the loss modulus over the tested frequency range, indicating an elastic-dominant response associated with a particle-supported internal network. F1_Sm showed consistently higher complex viscosity and higher G′ and G″ values than F2_Sr, suggesting stronger particle–particle and/or particle–binder interactions. The lower viscosity and moduli of F2_Sr indicate easier flow but a weaker internal network; the low-frequency changes observed at higher temperature are consistent with network relaxation and local rearrangement within the less homogeneous SFO-based feedstock. These temperature-dependent trends confirm that both feedstocks remained within a usable processing range for printing with a common nozzle temperature of 220–230 °C and a 0.8 mm nozzle.

The successful fabrication of ring-shaped specimens confirms the practical relevance of the rheological results. Both feedstocks could be printed using the same process window, producing structurally continuous parts with no large internal defects visible by SEM or micro-CT. Previous studies on MEX have shown that stable extrusion and interlayer bonding depend on the combined effects of melt flow, thermal history, contact pressure, and strand stability after deposition [16,17,21,22]. In the present work, the use of a mildly heated chamber likely reduced thermal gradients and supported interlayer contact during deposition, while the selected layer height and printing speed helped maintain dimensional stability. The local material accumulation observed near the start region of the printed rings is consistent with the sensitivity of MEX parts to toolpath transitions, local velocity changes, and transient extrusion behavior during path initiation and termination.

The magnetic measurements showed that the printed specimens retained hard-magnetic behavior after filament extrusion and MEX printing. Because the VSM data were normalized by total sample mass, the decrease in remanent magnetization, M_r_, from powder to filament is mainly attributed to dilution by the non-magnetic binder. The smaller additional decrease from filament to printed ring may be associated with processing-related changes during nozzle extrusion and strand deposition, including local particle rearrangement, density variation, or differences in sample geometry. The observed decrease in H_c_ from powder to filament to printed ring should be interpreted cautiously, because coercivity is not directly controlled by binder dilution. Instead, it may reflect changes in particle interactions, local surface conditions, demagnetizing effects, or processing-induced modifications during compounding and printing. Importantly, both materials preserved their overall hard-magnetic character after printing.

Printed magnetic polymer composites are attractive for customized magnetic assemblies, sensors, actuator components, lightweight functional fixtures, prototype magnetic circuits, and application-specific ring or complex geometries where design freedom is important [4,6,23]. Compared with classic sintered parts, MEX-printed bonded magnetic composites offer lower-temperature shaping, reduced tooling requirements, rapid design iteration, and the ability to produce complex geometries directly from filament feedstocks [4,6,24]. However, sintered magnets generally retain advantages in density, magnetic powder fraction, and maximum magnetic performance [25]. Therefore, the benefit of the present approach is not to replace all sintered magnets, but to provide a flexible manufacturing route for geometrically complex or customized bonded magnets where processability and design freedom are central requirements.

Overall, this work demonstrates that a TPE + gPO polyolefin-based binder can provide a suitable processing window for highly filled SmFeN and SFO magnetic feedstocks. The binder enabled filament extrusion, automatic spooling, and MEX printing while maintaining sufficient mechanical integrity and favorable rheological behavior. Compared with previous studies on polymer-bonded magnetic filaments and highly filled MEX feedstocks [1,4,17,19,22], the present results highlight the importance of combining rheological analysis with microstructural and magnetic characterization. Future work should focus on field-assisted deposition or post-print magnetic alignment, further optimization of powder loading and binder composition, and quantitative assessment of magnetic anisotropy.

## 5. Conclusions

- A TPE + gPO polyolefin-based binder enabled the preparation of highly filled SmFeN- and SFO-based feedstocks with residual powder contents of approximately 89 wt.% and 85 wt.%, respectively.
- Both feedstocks were extruded into dimensionally stable 2.85 mm filaments that could be automatically spooled and handled for MEX processing.
- The feedstocks exhibited pronounced shear-thinning behavior and elastic-dominant viscoelasticity, supporting flow through the nozzle and strand stability after deposition.
- Mechanical, SEM, and micro-CT results showed that the SmFeN-based filament had a more homogeneous particle–binder network and higher stiffness, while the SFO-based filament showed easier flow but more local heterogeneity.
- Fractographic observations confirm brittle-dominated tensile failure in both composites, with localized filler–matrix debonding or particle pull-out but no evidence of extensive magnetic filler delamination.
- Both materials were successfully printed into structurally continuous ring-shaped bonded magnets that retained hard-magnetic behavior after MEX processing.
- The results demonstrate that rheology-guided binder design is a useful route for developing highly filled magnetic filaments for geometry-flexible bonded magnets.

## Figures and Tables

**Table 1:** Particle size distribution, density, and magnetic properties of the starting magnetic powders.

Filler Material	Particle Size Distribution				Density (g/cm^3^)	Coercivity, H_c_ (kA/m)	Remanent Magnetization, M_r_ (emu/g)
	D10 (µm)	D50 (µm)	D90 (µm)	D90/D10 Ratio (µm)			
SmFeN	2.05	4.04	8.01	4.39	7.2	1110	78
SFO	1.28	3.12	6.56	5.11	5.1	296	36

**Table 2:** Composition and compounding parameters of the SmFeN- and SFO-based feedstocks.

Feedstock	Filler Powder	Powder Content (vol.%)	Powder Content (wt.%)	TPE (wt.%)	gPO (wt.%)	Compounding Temperature (°C)	Speed (rpm)	Total Kneading Time (min)
F1_Sm	SmFeN	50	89.28	7.13	3.59	200	30–60	45
F2_Sr	SFO	50	84.89	10.06	5.05	200	30–60	45

**Table 3:** Filament extrusion parameters and measured filament properties.

Filaments	Extrusion Temp. (°C)	Die Temperature (°C)	Screw Speed (rpm)	Diameter (mm)	Ovality (mm)	Density (g/cm^3^)
F1_Sm	180–190	190	54	2.85 ± 0.07	0.017 ± 0.01	4.25
F2_Sr	160–170	170	54	2.85 ± 0.05	0.016 ± 0.009	3.10

**Table 4:** Mechanical properties of the extruded feedstock filaments F1_Sm and F2_Sr.

	Bending Properties		Tensile Properties	
Material	Maximum Flexural Stress (MPa)	Flexural Modulus (GPa)	UTS (MPa)	Young Modulus (GPa)
F1_Sm	31.45 ± 1.77	5.5 ± 0.2	11.40 ± 0.73	2.1 ± 0.01
F2_Sr	36.12 ± 0.14	3.0 ± 0.04	12.28 ± 0.39	1.7 ± 0.03

**Table 5:** MEX printing parameters used for the fabrication of ring-shaped specimens.

Printing Parameters	Values
Nozzle temperature (°C)	220–230
Bed temperature (°C)	25 °C
Chamber temperature (°C)	55–60
Printing speed (mm/s)	5–11
Layer height (mm)	0.20–0.30
Extrusion multiplier (%)	0.90–1.05
Infill density (%)	100
Infill orientation	±45°

**Table 6:** Density and VSM-derived magnetic properties of extruded filaments and MEX-printed ring specimens.

Filler Material	Density (g cm^−3^)	Coercivity (kA/m)	Remanent Magnetization, M_r_ (emu/g)
F1_Sm R1_Sm	4.27 4.36	950 863	62 60
F2_Sr R2_Sr	3.1 3.2	278 264	32 26

## Data Availability

The original contributions presented in this study are included in the article. Further inquiries can be directed to the corresponding author.

## References

[1] Li L., Post B., Kunc V., Elliott A.M., Paranthaman M.P. (2017). Additive manufacturing of near-net-shape bonded magnets: Prospects and challenges. Scr. Mater..

[2] Huber C., Abert C., Bruckner F., Pfaff C., Kriwet J., Groenefeld M., Teliban I., Vogler C., Suess D. (2017). Topology optimized and 3D printed polymer-bonded permanent magnets for a predefined external field. J. Appl. Phys..

[3] Sonnleitner K., Huber C., Teliban I., Kobe S., Saje B., Kagerbauer D., Reissner M., Lengauer C., Groenefeld M., Suess D. (2020). 3D printing of polymer-bonded anisotropic magnets in an external magnetic field and by a modified production process. Appl. Phys. Lett..

[4] Huber C., Cano S., Teliban I., Schuschnigg S., Groenefeld M., Suess D. (2020). Polymer-bonded anisotropic SrFe_12_O_19_ filaments for fused filament fabrication. J. Appl. Phys..

[5] Li L., Tirado A., Nlebedim I.C., Rios O., Post B., Kunc V., Lowden R.R., Lara-Curzio E., Fredette R., Ormerod J. (2016). Big Area Additive Manufacturing of High Performance Bonded NdFeB Magnets. Sci. Rep..

[6] Von Petersdorff-Campen K., Hauswirth Y., Carpenter J., Hagmann A., Boës S., Schmid Daners M., Penner D., Meboldt M. (2018). 3D Printing of Functional Assemblies with Integrated Polymer-Bonded Magnets Demonstrated with a Prototype of a Rotary Blood Pump. Appl. Sci..

[7] Paranthaman M.P., Shafer C.S., Elliott A.M., Siddel D.H., McGuire M.A., Springfield R.M., Martin J., Fredette R., Ormerod J. (2016). Binder Jetting: A Novel NdFeB Bonded Magnet Fabrication Process. JOM.

[8] Huber C., Mitteramskogler G., Goertler M., Teliban I., Groenefeld M., Suess D. (2020). Additive Manufactured Polymer-Bonded Isotropic NdFeB Magnets by Stereolithography and Their Comparison to Fused Filament Fabricated and Selective Laser Sintered Magnets. Materials.

[9] Mapley M.C., Tansley G., Pauls J.P., Gregory S.D., Busch A. (2021). Selective laser sintering of bonded anisotropic permanent magnets using an in situ alignment fixture. Rapid Prototyp. J..

[10] Compton B.G., Kemp J.W., Novikov T.V., Pack R.C., Nlebedim C.I., Duty C.E., Rios O., Paranthaman M.P. (2018). Direct-write 3D printing of NdFeB bonded magnets. Mater. Manuf. Processes.

[11] Huber C., Abert C., Bruckner F., Groenefeld M., Muthsam O., Schuschnigg S., Sirak K., Thanhoffer R., Teliban I., Vogler C. (2016). 3D print of polymer bonded rare-earth magnets, and 3D magnetic field scanning with an end-user 3D printer. Appl. Phys. Lett..

[12] Geng J., Nlebedim I.C., Besser M.F., Simsek E., Ott R.T. (2016). Bulk Combinatorial Synthesis and High Throughput Characterization for Rapid Assessment of Magnetic Materials: Application of Laser Engineered Net Shaping (LENS™). JOM.

[13] Gonzalez-Gutierrez J., Cano S., Schuschnigg S., Kukla C., Sapkota J., Holzer C. (2018). Additive Manufacturing of Metallic and Ceramic Components by the Material Extrusion of Highly-Filled Polymers: A Review and Future Perspectives. Materials.

[14] Santos C., Gatões D., Cerejo F., Vieira M.T. (2021). Influence of Metallic Powder Characteristics on Extruded Feedstock Performance for Indirect Additive Manufacturing. Materials.

[15] Sadaf M., Cano S., Bragaglia M., Schuschnigg S., Kukla C., Holzer C., Vály L., Kitzmantel M., Nanni F., Gonzalez-Gutierrez J. (2024). Comparative analysis of binder systems in copper feedstocks for metal extrusion additive manufacturing and metal injection moulding. J. Mater. Res. Technol..

[16] Sadaf M., Cano S., Gonzalez-Gutierrez J., Bragaglia M., Schuschnigg S., Kukla C., Holzer C., Vály L., Kitzmantel M., Nanni F. (2022). Influence of Binder Composition and Material Extrusion (MEX) Parameters on the 3D Printing of Highly Filled Copper Feedstocks. Polymers.

[17] Kim S.H., Kim S.J., Park S.J., Mun J.H., Kang T.G., Park J.M. (2012). Rheological behavior of magnetic powder mixtures for magnetic PIM. Korea-Aust. Rheol. J..

[18] Chinnakorn A., Parsompech N., Suwanpreecha C., Suwannak P., Singkronart K., Schuschnigg S., Kukla C., Hararak B., Manonukul A. (2025). Evaluation of polymeric binders in 316L stainless steel manufacturing by filament-based MEX. Prog. Addit. Manuf..

[19] Cerejo F., Gatões D., Vieira M.T. (2021). Optimization of metallic powder filaments for additive manufacturing extrusion (MEX). Int. J. Adv. Manuf. Tech..

[20] Sadaf M., Bragaglia M., Nanni F. (2021). A simple route for additive manufacturing of 316L stainless steel via Fused Filament Fabrication. J. Manuf. Process..

[21] Sadaf M., Bragaglia M., Slemenik Perše L., Nanni F. (2024). Advancements in Metal Additive Manufacturing: A Comprehensive Review of Material Extrusion with Highly Filled Polymers. J. Manuf. Mater. Process..

[22] Arrigo R., Frache A. (2022). FDM Printability of PLA Based-Materials: The Key Role of the Rheological Behavior. Polymers.

[23] Ehrmann G., Blachowicz T., Ehrmann A. (2022). Magnetic 3D-Printed Composites—Production and Applications. Polymers.

[24] Hwang J.Y., Park S.J., Son Y., Jung H.Y. (2023). Influence of In Situ Magnetic Field on Magnetic Properties of a Bonded Permanent Magnet Manufactured through Material Extrusion Additive Manufacturing. Metals.

[25] Cui J., Ormerod J., Parker D., Ott R., Palasyuk A., McCall S., Paranthaman M.P., Kesler M.S., McGuire M.A., Nlebedim I.C. (2022). Manufacturing Processes for Permanent Magnets: Part I—Sintering and Casting. JOM.

